# Modifiable factors associated with frailty among indigenous older adults in southern Taiwan: a community-based cross-sectional study

**DOI:** 10.3389/fpubh.2026.1941122

**Published:** 2026-09-08

**Authors:** Aih-Fung Chiu, Chun-Fung Chiu, Chun-Man Hsieh

**Affiliations:** 1 Department of Nursing, Cheng Shiu University, Kaohsiung, Taiwan; 2 Department of Nursing, Kaohsiung Veterans General Hospital, Kaohsiung, Taiwan; 3 Department of Nursing, Tajen University, Yanpu, Pingtung, Taiwan

**Keywords:** frailty, gait speed, healthy aging, indigenous older adults, physical activity, Taiwan

## Abstract

**Background:**

Frailty is a major public health challenge among older adults. Indigenous populations experience poorer health outcomes and a greater burden of frailty than the general population. However, evidence on modifiable factors associated with frailty in Taiwanese Indigenous older adults remains limited. This study examined the associations between nutritional status, anthropometric characteristics, physical function, physical activity, and frailty among Indigenous older adults living in rural communities.

**Methods:**

A community-based, cross-sectional study was conducted among 117 Indigenous older adults aged ≥55 years attending Indigenous Cultural Health Stations. As the outcome measure, frailty was assessed using the Chinese version of the Clinical Frailty Scale (CFS-C). Predictor variables included nutritional status measured using the Mini Nutritional Assessment–Short Form, anthropometric characteristics (body mass index and calf circumference), physical function (handgrip strength and preferred gait speed), and physical activity assessed using the International Physical Activity Questionnaire–Short Form. Multivariable binary logistic regression was performed to identify factors independently associated with frailty.

**Results:**

Of the 117 participants, 27 (23.1%) were classified as frail (CFS-C ≥ 4). Compared with non-frail participants, frail participants were older and had slower preferred gait speed, weaker handgrip strength, and lower physical activity levels (all *p* < 0.01). After adjustment for age, sex, handgrip strength, preferred gait speed, and physical activity, faster preferred gait speed (OR = 0.266, 95% CI = 0.139–0.510, *p* < 0.001) and higher physical activity (OR = 0.998, 95% CI = 0.996–0.999, *p* = 0.007) remained independently associated with lower odds of frailty.

**Conclusion:**

Preferred gait speed and physical activity were independently associated with lower odds of frailty among Indigenous older adults. Routine gait speed assessment and attention to physical activity in Indigenous Cultural Health Stations may support frailty identification and community-based health promotion.

## 1 Introduction

With the rapid aging of populations worldwide, frailty has emerged as a major public health concern among older adults (1). Frailty is increasingly recognized as a state of reduced physiological reserve and resilience, resulting in heightened vulnerability to internal and external stressors (2–4). Previous studies have shown that frailty is associated with a range of adverse health outcomes among older adults, including disability, hospitalization, institutionalization, and mortality (4). Given its considerable health burden and adverse consequences, identifying the factors associated with frailty is essential for developing effective prevention and intervention strategies.

Although frailty is common among community-dwelling older adults, prevalence estimates vary substantially, depending on the assessment instrument used. A systematic review reported a pooled prevalence of physical frailty of 9.9% among community-dwelling older adults (5). Available evidence from Taiwan indicates that the prevalence of frailty among community-dwelling older adults ranges from approximately 4.9 to 14.9%, depending on the study population, frailty assessment method, and operational definition of frailty (6, 7). Given the high prevalence of frailty and its adverse health consequences, preventing frailty and promoting healthy ageing have become important public health priorities.

Current evidence suggests that Indigenous populations experience frailty at younger ages and with higher prevalence than non-Indigenous populations. However, research on culturally appropriate frailty assessment and intervention remains scarce (8). This disproportionate burden is likely attributable to the cumulative effects of lifelong social, economic, and environmental adversity rather than Indigenous identity *per se* (9). Consistent with this perspective, a recent scoping review in Taiwan highlighted that Indigenous peoples experience substantial health disparities, including higher mortality rates, a greater burden of infectious and chronic diseases, and limited access to healthcare resources, preventive services, and health promotion programs. These inequities are driven by the complex interplay of social, economic, cultural, and environmental determinants (10). These long-standing inequities may contribute to the disproportionately higher burden of frailty observed among Indigenous populations. Therefore, identifying modifiable factors associated with frailty is essential for informing early community-based screening and developing targeted prevention strategies, particularly in Indigenous communities affected by persistent health inequities and geographic barriers to healthcare.

Frailty is a multifactorial syndrome shaped by both non-modifiable and modifiable factors. Older age, female sex, and multimorbidity are well-established, non-modifiable factors associated with frailty (1, 2, 11). Among the modifiable factors, nutritional status, anthropometric characteristics, physical function, and physical activity have consistently been associated with frailty and, therefore, represent important targets for prevention. Malnutrition, as identified using the Mini Nutritional Assessment (MNA) or the Mini Nutritional Assessment–Short Form (MNA-SF), has consistently been associated with frailty (12, 13). A systematic review and meta-analysis found that both obesity and underweight, as measured by body mass index (BMI), were associated with an increased risk of frailty among community-dwelling older adults (14). Calf circumference, a practical surrogate marker of skeletal muscle mass, has also been linked to a lower risk of frailty and better physical function among community-dwelling older adults (15).

Handgrip strength and gait speed are widely recognized indicators of physical function in older adults. Both the physical frailty phenotype proposed by Fried et al. and the 2025 Asian Working Group for Sarcopenia (AWGS) consensus recognize handgrip strength and gait speed as essential indicators of muscle health, representing muscle strength and physical performance, respectively (16, 17).

Physical activity is another important modifiable factor for frailty. Insufficient physical activity has consistently been associated with an increased risk of frailty, whereas regular participation in moderate- to vigorous-intensity physical activity reduces frailty risk and delays its progression among older adults (2, 3, 18). Taken together, these modifiable factors constitute practical and actionable targets for community-based frailty screening, health promotion, and prevention, particularly in resource-limited Indigenous communities.

Despite increasing recognition of health disparities among Indigenous peoples, evidence regarding the factors associated with frailty among Taiwanese Indigenous older adults remains limited, particularly regarding modifiable factors. Most previous studies have focused on the general older population; therefore, it remains unclear whether these established modifiable factors are similarly associated with frailty among Taiwanese Indigenous older adults. Addressing this knowledge gap may enable the early identification of frailty and provide evidence to inform culturally appropriate public health strategies, community-based screening, and preventive interventions for Indigenous older adults. Accordingly, this study aimed to identify the factors associated with frailty among Indigenous older adults residing in rural communities in southern Taiwan. Specifically, this study examined the associations between frailty and nutritional status, body mass index, calf circumference, handgrip strength, gait speed, and physical activity among Indigenous older adults.

## 2 Method

### 2.1 Study design, setting, and participants

This was a community-based cross-sectional study using purposive sampling. The study population consisted of community-dwelling Indigenous older adults living in rural communities in southern Taiwan. Participants were recruited through the seven Indigenous Cultural Health Stations, which are community-based centers providing health promotion and supportive services for Indigenous older adults. Participants were not required to be current users of Cultural Health Station services. Eligible participants were Indigenous adults aged ≥55 years who were registered residents of the study communities, were able to ambulate independently with or without assistive devices, were able to communicate in Mandarin, Taiwanese, Hakka, or an Indigenous language with the assistance of an interpreter, and provided written informed consent before participation. The age threshold of ≥55 years was adopted in accordance with Taiwan’s long-term care policy, which uses 55 years as an eligibility threshold for Indigenous populations (19). Individuals who did not meet the inclusion criteria were not enrolled. Of the 122 eligible individuals, two were unable to complete the study because of time constraints, while three did not complete all required study procedures and therefore had incomplete data. Ultimately, 117 participants were included in the final analyses.

### 2.2 Data collection

Data were collected between October and November 2022 through face-to-face interviews conducted by trained research personnel. Before participant recruitment, all research personnel received standardized training on the study protocol and assessment procedures to ensure consistency in data collection. Permission to conduct the study was obtained from the Council of Indigenous Peoples, and the seven participating Indigenous Cultural Health Stations were subsequently contacted to explain the study objectives and recruitment procedures and facilitate participant recruitment. Written informed consent was obtained from all participants before data collection. Participants first completed structured questionnaires to obtain demographic characteristics, clinical characteristics, nutritional status, and physical activity information. Frailty status, anthropometric measurements (height, weight, and calf circumference), and physical function measures (handgrip strength and preferred gait speed) were subsequently assessed according to standardized protocols. All assessments were completed on the same day. The study was approved by the Ethics Committee of National Cheng Kung University (NCKU HREC-E-110-488-2) and conducted in accordance with the Declaration of Helsinki.

### 2.3 Outcome measure: frailty

Frailty status was assessed using the Chinese version of the Clinical Frailty Scale (CFS-C). The Clinical Frailty Scale (CFS), originally developed by Rockwood and colleagues as part of the Canadian Study of Health and Aging, is a validated clinical instrument for assessing frailty. The CFS is strongly correlated with the Frailty Index and independently predicts mortality and institutionalization among older adults (20). The CFS-C is a nine-point scale ranging from 1 (very fit) to 9 (terminally ill), with higher scores indicating greater frailty. The Chinese version has shown acceptable reliability and validity among older adults in Taiwan (21). For the primary analyses, frailty was dichotomized using a CFS-C score of 4 (vulnerable) as the cut-off. Participants with CFS-C scores of 1–3 were classified as non-frail, whereas those with scores of 4–9 were classified as frail.

### 2.4 Predictor variables

#### 2.4.1 Demographic and clinical characteristics

Demographic characteristics included age, sex, and living arrangement (living alone or not). Clinical characteristics comprised self-reported physician-diagnosed medical conditions, including hypertension, diabetes mellitus, heart disease, and chronic lung disease, as well as a history of falls in the previous year.

#### 2.4.2 Nutritional status

Nutritional status was assessed using the MNA-SF, a validated screening instrument for identifying malnutrition or the risk of malnutrition in older adults. The MNA-SF comprises six items that assess recent food intake, weight loss, mobility, psychological stress or acute disease, neuropsychological problems, and BMI. The total score ranged from 0 to 14. In this study, participants with MNA-SF scores <12 were classified as being at risk of malnutrition or malnourished, according to the developers’ recommended cut-off values (22, 23).

#### 2.4.3 Anthropometric measurements

Anthropometric measurements were conducted by trained research personnel using standardized procedures. Body weight and height were measured to calculate BMI in kg/m^2^. Participants were classified as underweight or normal weight (BMI < 24 kg/m^2^), overweight (BMI 24–<27 kg/m^2^), or obese (BMI ≥ 27 kg/m^2^) according to the criteria established by the Health Promotion Administration, Ministry of Health and Welfare, Taiwan. Calf circumference was measured at the point of maximal calf circumference using a non-elastic measuring tape, with participants seated in a relaxed position. According to the AWGS 2019 consensus, low calf circumference was defined as <34 cm for men and <33 cm for women (24).

#### 2.4.4 Physical function

Handgrip strength was measured using a digital hand dynamometer (TTM-YD, Tokyo, Japan), following a standardized protocol (25). Participants performed three maximal voluntary contractions with their dominant hand, with a one-minute rest interval between trials, and the highest value was used for analysis. Low handgrip strength was defined as <28 kg for men and <18 kg for women, according to the AWGS 2019 consensus (24).

Preferred gait speed was assessed using a standardized protocol (26). Participants were instructed to walk at their usual pace along a 6-m walkway. Participants who routinely used walking aids were permitted to use their usual assistive devices during the assessment. The time required to complete the walk was recorded with a stopwatch, and gait speed was calculated by dividing the walking distance by the elapsed time (m/s). Slow gait speed was defined as <1.0 m/s, according to the AWGS 2019 consensus (24).

#### 2.4.5 Physical activity

Physical activity was assessed using the Taiwan version of the International Physical Activity Questionnaire–Short Form (IPAQ-SF), which has demonstrated acceptable reliability and validity in Taiwanese adults (27). The IPAQ-SF assesses the frequency and duration of walking, moderate-intensity physical activity, and vigorous-intensity physical activity undertaken during the previous 7 days, provided that each activity lasted at least 10 consecutive minutes (28). Total physical activity was calculated as metabolic equivalent of task (MET)-minutes per week according to the IPAQ scoring protocol. Participants were classified into three physical activity categories according to the IPAQ scoring protocol: low (<600 MET-min/week), moderate (600 to <3,000 MET-min/week), and high (≥3,000 MET-min/week) (27, 29).

### 2.5 Statistical analysis

Participant characteristics were summarized using descriptive statistics. Categorical variables were presented as frequencies and percentages, and continuous variables as means and standard deviations (SDs). For descriptive analyses, continuous variables were categorized using established clinical or guideline-based cut-off values where appropriate. Differences between the non-frail and frail groups were assessed using the chi-square (*χ*^2^) test for categorical variables and the independent-samples *t* test for continuous variables. Variables that were statistically significant in the univariable analyses were considered for inclusion in the multivariable logistic regression model. When both continuous and categorized forms of the same variable were available, the continuous measure was entered into the multivariable model to preserve statistical power and minimize information loss due to categorization. Preferred gait speed was modeled per 0.1 m/s increase to facilitate clinical interpretation. Age and sex were included *a priori* based on their clinical relevance. Adjusted odds ratios (ORs) and 95% confidence intervals (CIs) were reported. Model fit was assessed using the Hosmer–Lemeshow goodness-of-fit test and the Cox and Snell and Nagelkerke pseudo-*R*^2^ statistics. Statistical analyses were performed using IBM SPSS Statistics version 23.0 (IBM Corp., Armonk, NY, USA). All tests were two-sided, and a *p* value < 0.05 was considered statistically significant.

## 3 Results

### 3.1 Demographic, clinical characteristics, and distribution of frailty status

As shown in Table 1, the 117 participants had a mean age of 73.4 ± 8.9 years, and most were women (76.1%). Hypertension (84.6%) and diabetes mellitus (34.2%) were the most prevalent chronic conditions. The mean body mass index was 29.7 ± 5.0 kg/m^2^, with 70.1% classified as obese. The mean preferred gait speed was 0.9 ± 0.3 m/s, and 61.5% exhibited slow gait speed (<1.0 m/s). Mean handgrip strength was 21.4 ± 5.8 kg, and the mean total physical activity was 1378.3 ± 1467.7 MET-min/week; 43.6% had low physical activity (<600 MET-min/week). The most common CFS-C score was 3 (53.8%), followed by scores of 2 (23.1%), 4 (19.7%), and 5 (3.4%). Overall, 27 participants (23.1%) met the criteria for CFS-C ≥ 4.

**Table 1:** Characteristics of indigenous older adults participating in the study (*N* = 117).

Variables	*n* (%) or mean ± SD
Sociodemographic characteristics	
Age, years (mean ± SD)	73.4 ± 8.9
55 to <65 years	18 (15.4)
65 to <75 years	44 (37.6)
75 to <85 years	44 (37.6)
≥85 years	11 (9.4)
Sex	
Men	28 (23.9)
Women	89 (76.1)
Living alone	18 (15.4)
Clinical conditions, *n* (%)	
Hypertension	99 (84.6)
Diabetes mellitus	40 (34.2)
Heart disease	10 (8.5)
Chronic lung disease	12 (10.3)
Falls in the previous year	22 (18.8)
Being at risk of malnutrition (MNA < 12)	9 (7.7)
Anthropometric characteristics	
Body mass index, kg/m^2^	29.7 ± 5.0
<24, *n* (%)	14 (12.0)
24–<27, *n* (%)	21 (17.9)
≥27, *n* (%)	82 (70.1)
Calf circumference, cm	36.1 ± 3.8
Men: Low calf circumference (< 34 cm), *n* (%)	6 (21.4)
Women: Low calf circumference (< 33 cm), *n* (%)	15 (16.9)
Physical function	
Preferred gait speed, m/s	0.9 ± 0.3
Slow gait speed (<1.0 m/s), *n* (%)	72 (61.5)
Handgrip strength, kg	21.4 ± 5.8
Men: Low handgrip strength (< 28 kg), *n* (%)	16 (57.1)
Women: Low handgrip strength (< 18 kg), *n* (%)	34 (38.2)
Physical activity	
Total physical activity, MET-min/week	1378.3 ± 1467.7
Low (<600 MET-min/week)	51 (43.6)
Moderate (≥600 - < 3,000 MET-min/week)	46 (39.3)
High (≥3,000 MET-min/week)	20 (17.1)
CFS-C scores	
2	27 (23.1)
3	63 (53.8)
4	23 (19.7)
5	4 (3.4)

Data are presented as mean ± standard deviation (SD) or *n* (%). MNA, Mini Nutritional Assessment; MET, metabolic equivalent task; CFS-C, Chinese version of the Clinical Frailty Scale.

### 3.2 Comparison between non-frail and frail participants

As shown in Table 2, frail participants were significantly older and had poorer physical function and lower physical activity than non-frail participants. Specifically, frail participants had slower preferred gait speed (0.54 ± 0.17 vs. 0.97 ± 0.22 m/s, *p* < 0.001), lower handgrip strength (18.35 ± 3.36 vs. 22.27 ± 6.07 kg, *p* = 0.002), and lower total physical activity (428.6 ± 526.2 vs. 1663.2 ± 1539.8 MET-min/week, *p* < 0.001). No significant differences were observed in sociodemographic characteristics, chronic diseases, nutritional risk, body mass index, or calf circumference (all *p* > 0.05).

**Table 2:** Participant characteristics by frailty status (*N* = 117).

Variables	Non-frail (*n* = 90)	Frail (*n* = 27)	*p* value
Sex, *n* (%)			
Men, *n* (%)	25 (27.8)	3 (11.1)	0.075
Women, *n* (%)	65 (72.2)	24 (88.9)	
Age, years	71.9 ± 8.7	78.4 ± 7.7	0.001*
Living alone	15 (16.7)	3 (11.1)	0.761
Hypertension, *n* (%)	79 (87.8)	20 (74.1)	0.125
Diabetes mellitus, *n* (%)	34 (37.8)	6 (22.2)	0.135
Heart disease, *n* (%)	7 (7.8)	3 (11.1)	0.695
Chronic lung disease, *n* (%)	8 (8.9)	4 (14.8)	0.468
Falls in the previous year, *n* (%)	16 (17.8)	6 (22.2)	0.604
Being at risk of malnutrition (MNA < 12)	8 (8.9)	1 (3.7)	0.682
Body mass index, kg/m^2^	29.8 ± 4.9	29.4 ± 5.4	0.739
<24, *n* (%)	11 (12.2)	3 (11.1)	0.986
24–<27, *n* (%)	16 (17.8)	5 (18.5)	
≥27, *n* (%)	63 (70.0)	19 (70.4)	
Calf circumference, cm	36.2 ± 3.8	35.8 ± 3.8	0.636
Men: Low calf circumference (<34 cm), n (%)	5 (20.0)	1 (33.3)	0.530
Women: Low calf circumference (<33 cm), n (%)	10 (15.4)	5 (20.8)	0.537
Preferred gait speed, m/s	1.0 ± 0.2	0.5 ± 0.2	<0.001*
Low preferred gait speed (<1 m/s), n (%)	45 (50.0)	27 (100.0)	<0.001*
Handgrip strength, kg	22.3 ± 6.1	18.4 ± 3.4	0.002*
Men: Low handgrip strength (<28 kg), n (%)	13 (52.0)	3 (100)	0.238
Women: Low handgrip strength (<18 kg), n (%)	19 (29.2)	15 (62.5)	0.004*
Total physical activity, MET-min/week	1663.2 ± 1539.8	428.6 ± 526.2	<0.001*
Low (<600 MET-min/week)	30 (33.3)	21 (77.8)	<0.001*
Moderate (≥600 - < 3,000 MET-min/week)	40 (44.4)	6 (22.2)	
High (≥3,000 MET-min/week)	20 (22.2)	0 (0.0)	

CFS-C, Chinese version of the Clinical Frailty Scale. Frailty was defined as a CFS-C score of ≥4. Group differences were assessed using the chi-square (*χ*^2^) test for categorical variables and the independent-samples *t* test for continuous variables. *p* < 0.05. The symbol * indicates statistical significance at *p* < 0.05.

### 3.3 Factors associated with frailty

As shown in Table 3, after adjustment for age, sex, handgrip strength, preferred gait speed, and total physical activity, only preferred gait speed and total physical activity remained independently associated with frailty. Higher gait speed (per 0.1 m/s increase; OR = 0.266, 95% CI = 0.139–0.510, *p* < 0.001) and greater total physical activity (OR = 0.998, 95% CI = 0.996–0.999, *p* = 0.007) were associated with lower odds of frailty. Age, sex, and handgrip strength were not independently associated with frailty. The model showed good calibration (Hosmer–Lemeshow *p* = 0.852) and acceptable explanatory power (Nagelkerke *R*^2^ = 0.760).

**Table 3:** Multivariable logistic regression for factors associated with frailty (*N* = 117).

Variables	OR	95% CI	*p* value
Age, years	1.030	0.917–1.156	0.618
Female sex (ref: male)	13.920	0.969–199.982	0.053
Handgrip strength, kg	1.021	0.817–1.277	0.853
Preferred gait speed (per 0.1 m/s increase)	0.266	0.139–0.510	<0.001*
Total physical activity, MET-min/week	0.998	0.996–0.999	0.007*

Binary logistic regression using the enter method was performed to examine modifiable factors associated with frailty. ^*^p < 0.05. Model fit statistics.

Hosmer–Lemeshow goodness-of-fit test: *χ*^2^ = 4.055, df = 8, *p* = 0.852; Cox and Snell *R*^2^ = 0.509, Nagelkerke *R*^2^ = 0.760.

OR, odds ratio; CI, confidence interval; CFS-C, Chinese version of the Clinical Frailty Scale. Frailty was defined as a CFS-C score of ≥4. Sex was coded with male as the reference category; all other predictors were entered as continuous variables, with preferred gait speed modeled per 0.1 m/s increase.

## 4 Discussion

The present study identified preferred gait speed and physical activity as factors independently associated with frailty among Indigenous older adults after adjustment for potential confounders, whereas age, sex, and handgrip strength were not independently associated. These findings suggest that functional performance and habitual physical activity may be more proximal indicators of frailty than chronological age or isolated measures of muscle strength.

Preferred gait speed was independently associated with frailty among Indigenous older adults. The present findings are consistent with the existing literature, demonstrating that slower gait speed is associated with an increased risk of disability, frailty, hospitalization, and mortality among older adults (30). Gait speed is widely recognized as a robust indicator of overall physiological reserve and functional capacity and has been proposed as the “sixth vital sign” because it reflects the integrated performance of multiple physiological systems, including the musculoskeletal, cardiovascular, respiratory, and nervous systems (26, 31). Given its low cost, non-invasive nature, feasibility in community settings, and ability to provide complementary objective information for frailty assessment, gait speed assessment may serve as a practical tool for routine frailty screening in Indigenous Cultural Health Stations, with potential relevance for the early identification of frailty and consideration of timely preventive interventions. Higher levels of physical activity were independently associated with a lower likelihood of frailty, even after adjustment for gait speed and other potential confounders. This finding is consistent with accumulating evidence linking higher levels of habitual physical activity with a lower risk of frailty. A recent systematic review reported that older adults with the highest levels of physical activity had a 41% lower risk of frailty than those with the lowest levels, regardless of the frailty definition or physical activity assessment method (32). Similarly, a prospective cohort study demonstrated that moderate- and vigorous-intensity physical activity was associated with a lower risk of frailty (20). These associations are biologically plausible: regular physical activity may help preserve physiological reserve and maintain physical function through its effects on muscle strength, balance, mobility, and cardiorespiratory fitness, while potentially attenuating age-related chronic inflammation and oxidative stress, which may in turn contribute to delaying the development of frailty (33). Given the geographical barriers and limited healthcare access in many Indigenous communities, community-based physical activity programs integrated into routine health promotion may be a relevant strategy for supporting health promotion.

Indigenous Cultural Health Stations may provide practical settings for brief gait speed assessment and culturally appropriate physical activity. Given the staffing and service demands of these community-based settings, together with the limited family support available to some older adults, intervention strategies should be simple and feasible to integrate into existing community activities rather than relying on resource-intensive individualized or home-based programs. A systematic review synthesized randomized controlled trial evidence indicating that exercise training may improve frailty (34), and the population-based findings of the present study highlight the potential relevance of such interventions for Indigenous communities. Nevertheless, these associations should be interpreted within the broader social and environmental context of Indigenous communities, including geographical conditions, ongoing engagement in farming and labor, community-based activities, and the social, cultural, and religious functions of Cultural Health Stations (35), all of which may influence older adults’ habitual physical activity. However, these contextual factors were not directly assessed in the present study, and their actual influence warrants further investigation.

Handgrip strength was significantly associated with frailty in the univariable analysis but was no longer independently associated with frailty after adjustment for age, sex, physical activity, and preferred gait speed. Although handgrip strength and preferred gait speed were weakly and positively correlated (Pearson’s r = 0.258, *p* < 0.01), variance inflation factor (VIF) values for all predictors ranged from 1.221 to 1.873, indicating the absence of problematic multicollinearity. Thus, the loss of statistical significance for handgrip strength was unlikely to be attributable to multicollinearity; rather, its unadjusted association with frailty may have been attenuated after accounting for demographic characteristics and other measures of physical function and activity. Moreover, because the CFS-C primarily reflects overall functional status and mobility, preferred gait speed and physical activity may capture frailty-related functional limitations more directly than handgrip strength. This interpretation is indirectly supported by a systematic review showing that, although both lower handgrip strength and slower gait speed were associated with cardiovascular mortality, the association was more consistent for gait speed than for handgrip strength (36). Nevertheless, this evidence should be interpreted cautiously because the systematic review examined cardiovascular mortality rather than frailty, and the number of frailty events in the present study was limited.

Neither nutritional status nor calf circumference was significantly associated with frailty in the univariable analysis, and these variables were therefore not included in the multivariable model. This finding contrasts with previous evidence. Earlier studies using the MNA or MNA-SF reported that malnutrition or risk of malnutrition was associated with frailty (12, 13, 37), and another study found that larger calf circumference was associated with a lower frailty index and better functional performance among community-dwelling older adults (15). The absence of significant associations in the present study may be partly attributable to the relatively small sample size (*n* = 117) and the limited number of participants with a low MNA-SF score (<12; *n* = 9, 7.7%) or a low calf circumference (men: <34 cm, *n* = 6/28, 21.4%; women: <33 cm, n = 15/89, 16.9%). These factors may have limited the statistical power to detect modest associations.

BMI was not significantly associated with frailty in either the univariable or multivariable analysis. This finding contrasts with a systematic review and meta-analysis showing that both obesity and underweight were associated with an increased risk of frailty among community-dwelling older adults, suggesting a U-shaped rather than a linear association (14). Two factors may account for this discrepancy. First, because 70.1% of participants in the present study were classified as obese (BMI ≥ 27 kg/m^2^), the restricted range of BMI values may have limited the statistical power to detect a U-shaped or otherwise nonlinear association. Second, BMI does not distinguish fat mass from lean mass and may therefore inadequately capture body composition. DXA-measured percentage body fat, rather than BMI, has been associated with gait speed maintenance in older women (38), suggesting that direct measures of body composition may provide information not reflected by BMI. However, because that study assessed gait speed rather than frailty, this evidence remains indirect. Taken together, these findings suggest that BMI alone may be insufficient to characterize frailty risk in populations with a high prevalence of obesity. These null findings should be interpreted with caution, and further research involving larger Indigenous populations and more comprehensive assessments of body composition is warranted.

To our knowledge, this is among the first studies in Taiwan to investigate potentially modifiable factors associated with frailty among Indigenous older adults using the validated CFS-C. These findings carry important clinical and public health implications, highlighting the potential relevance of gait speed and physical activity to frailty assessment and supporting their incorporation into routine screening at Indigenous Cultural Health Stations. Longitudinal studies are warranted to clarify the temporal and causal nature of these associations. Several limitations should be acknowledged. First, because of the cross-sectional design, causal inferences cannot be made. Reverse causality is also possible, as frailty itself may lead to slower gait speed and lower levels of physical activity. Longitudinal studies are therefore needed to clarify the temporal relationships among gait speed, physical activity, and frailty. Second, participants were recruited using purposive sampling from Indigenous Cultural Health Stations in southern Taiwan. This recruitment strategy, together with the relatively small sample size (*n* = 117) and predominance of women (76.1%), may limit the generalizability of the findings. The small number of male participants (*n* = 28), only three of whom were classified as frail, precluded adequately powered sex-stratified analyses. Previous longitudinal evidence suggests that factors associated with gait speed transitions may differ by sex (38). Future studies with larger and more sex-balanced samples are needed to examine potential sex-specific associations. Furthermore, the findings may not be applicable to Indigenous older adults who do not attend or have access to Cultural Health Stations, particularly homebound individuals, or to Indigenous populations in other regions of Taiwan. Information on eligible individuals who declined participation or were not enrolled was unavailable. Cross-study comparisons of frailty prevalence and associated factors should therefore be interpreted with caution. In addition, the inclusion of Indigenous adults aged ≥55 years, consistent with Taiwan’s long-term care policy, may limit direct comparability with national and international studies that define older populations using age thresholds of ≥60 or ≥65 years. Therefore, comparisons of frailty prevalence and associated factors across studies should be interpreted with caution. Third, physical activity was assessed using the self-reported IPAQ-SF, which is susceptible to recall bias. Finally, sedentary behavior was not assessed in this study. Given evidence that sedentary behavior is associated with frailty independently of physical activity (34, 39), the observed association between physical activity and frailty may also have been influenced by unmeasured sedentary behavior. Future studies should consider using objective measures, such as accelerometry, to assess both physical activity and sedentary behavior, thereby further clarifying their independent associations with frailty.

## 5 Conclusion

Among Indigenous older adults living in rural communities in southern Taiwan, preferred gait speed and physical activity were independently associated with frailty. These findings identify two practical, potentially modifiable targets for community-based frailty prevention. Incorporating routine gait speed assessment and promoting regular physical activity within Indigenous Cultural Health Stations may facilitate early frailty identification and support culturally appropriate prevention strategies. Future longitudinal studies are warranted to confirm these findings and evaluate interventions targeting these modifiable factors.

## Data Availability

The raw data supporting the conclusions of this article will be made available by the authors, without undue reservation.
